# Comparative Assessment of Growth and Quality Characteristics of Tea Nursery Plants Propagated by Seed and Cutting

**DOI:** 10.3390/plants15213249

**Published:** 2026-10-22

**Authors:** Gizem Öztürk, Keziban Yazıcı

**Affiliations:** 1 Department of Horticulture, Faculty of Agriculture, Recep Tayyip Erdoğan University, Rize 53300, Türkiye; keziban.yazici@erdogan.edu.tr; 2 Tea and Tea Products Application and Research Center, Recep Tayyip Erdoğan University, Rize 53300, Türkiye

**Keywords:** *Camellia sinensis* L., cutting propagation, seed propagation, nursery plant quality, root development, TeaRDS

## Abstract

Tea (*Camellia sinensis* L.) cultivation is an important agricultural activity in the Eastern Black Sea Region of Türkiye, where commercial tea production is concentrated. Most Turkish tea plantations were established from seed, resulting in genetic and phenotypic variability, while vegetative propagation remains limited. This study compared seed and cutting propagation across eight tea plant materials to evaluate their effects on nursery plant performance and the potential of clonal propagation for plantation renewal. For each genotype, 48 seeds and 48 leaf-bud cuttings were established, totaling 768 propagation units. To provide a more consistent assessment of root development, method-specific five-level tea root development scales (TeaRDS) were developed for seed- and cutting- derived nursery plants. Seed-derived nursery plants generally showed greater shoot growth, stem diameter, and root weight. In contrast, TeaRDS-based root development scores did not differ significantly between propagation methods. The effects of propagation method varied among genotypes, and multivariate analyses showed that cutting- and seed-derived plants had the most similar developmental profiles in 53 ME 265 and 53 AD 83 (d = 1.85 and 2.03, respectively). These findings demonstrate that, although cutting propagation does not provide the same growth performance as seed propagation in all genotypes, selected genotypes can achieve comparable developmental profiles, supporting the use of clonal propagation as a promising strategy for the renewal of aging tea plantations in Türkiye.

## 1. Introduction

The tea plant (*Camellia sinensis* L.) is one of the most widely consumed beverages worldwide and has considerable economic and social importance. It is cultivated mainly in China, India, Sri Lanka, Türkiye, and Southeast Asian countries, where it provides a source of income for millions of people [1]. Tea cultivation has been carried out for more than 100 years across extensive areas in the Eastern Black Sea Region of Türkiye. Following investigations conducted in Batumi in 1917, it was reported that the Eastern Black Sea Region of Türkiye was suitable for tea cultivation. The first tea plantations in Türkiye were established using tea seeds imported from Batumi, Georgia, and large-scale plantation establishment subsequently expanded with further seed imports during the late 1930s and early 1940s [2,3]. The tea industry experienced substantial growth after the 1990s, and the global tea cultivation area expanded by approximately 44% between 1991 and 2010 [4,5]. Today, Türkiye ranks fifth worldwide, with 1.3 million tons of fresh tea leaf production according to 2023 production data [6].

The yield declines and production fluctuations observed in recent years are associated with aging tea plantations, changing climatic conditions, and increasing environmental stress factors. Therefore, the renewal of tea plantations has become increasingly important for sustainable production. Indeed, studies revealing the negative effects of climate change on tea production in Türkiye also support this view [7]. The economic lifespan of tea plants is approximately 50–60 years, and rejuvenation pruning is practiced in Türkiye to maintain productivity. However, pruning and other cultural practices cannot fully compensate for yield and quality losses in plantations that have reached the end of their economic lifespan [8]. Yield reduction, quality deterioration, and increasing maintenance costs in aging tea plantations are among the major factors limiting sustainable tea production. Considering Türkiye’s approximately 100-year history of tea cultivation, a significant proportion of existing plantations require renewal. Therefore, the replacement of aging tea plantations with high-quality clonal nursery plants obtained from disease-resistant, high-yielding, and superior-quality genotypes could improve plantation sustainability and contribute to enhancing the resilience of tea cultivation to environmental pressures, including climate change [9,10].

In tea nursery plant production, propagation can be carried out generatively by seed and vegetatively by cuttings, grafting, and tissue culture. Propagation by cuttings has become one of the most widely used production methods due to its ease of application and the ability to obtain nursery plants in a relatively short time [8,11]. Cutting propagation enables the clonal multiplication of selected superior individuals. This approach can provide advantages by preserving desirable traits and enabling the establishment of genetically homogeneous plantations, thereby contributing to more uniform yield and quality [8,12]. Adventitious root formation is the fundamental process underlying cutting propagation, and various hormonal and environmental factors, particularly auxin, play decisive roles in this process [13,14,15]. Studies comparing the effectiveness of different rooting agents have generally shown that IBA is effective in promoting adventitious root formation [16,17]. The establishment of tea plantations from seed leads to the formation of genetically heterogeneous populations, resulting in considerable variation in yield, quality, and morphological traits. Such variation can complicate product standardization in commercial tea production and may undermine quality continuity [8,18]. Therefore, the adoption of vegetative, or clonal, propagation methods, which are standard practice in many major tea-producing countries, is of great importance for establishing genetically homogeneous, high-yielding, and high-quality plantations in modern tea cultivation [19].

Although seed propagation remains widely used in Türkiye, vegetative propagation is increasingly important in modern tea production systems [8]. For the renewal of aging tea plantations, the clonal propagation of selected superior genotypes with desirable yield and quality characteristics represents an important strategy for sustainable production [8,9]. Although studies on cutting propagation and seed propagation in tea are available in the literature, direct comparisons of these two propagation methods under the same growing conditions, particularly with respect to root and shoot development and shoot quality, remain limited. Therefore, this study aimed to compare seed- and cutting-derived nursery plants across eight tea plant materials in terms of propagation success, root development, shoot growth, and shoot quality, and to determine whether cutting-derived plants can achieve developmental performance comparable to that of seed-derived plants. The study further aimed to provide an evidence-based assessment of the potential use of clonal nursery plants for the renewal of aging tea plantations.

## 2. Materials and Methods

### 2.1. Plant Materials and Experimental Design

The study was conducted during 2024–2025 in a 500 m^2^ climate-controlled polycarbonate greenhouse used for tea research and nursery production, located at the National Tea Gene Pool of the Faculty of Agriculture, Recep Tayyip Erdoğan University. A total of eight tea (*Camellia sinensis* L.) plant materials, comprising three cultivars and five genotypes, were used in the study. The cultivars included CIN-1 of Chinese origin, SEAKARI of Japanese origin, and TUGLALI, a registered Turkish cultivar developed by ÇAYKUR, representing commercially important cultivars of different origins. The five Turkish genotypes (53 ME 265, 28 TI 09, 53 CE 279, 53 AD 83, and 53 GU 40) were selected from the RTEÜ National Tea Gene Pool, Rize, Türkiye, based on their distinct characteristics identified during previous evaluations conducted within a national research project supported by the Scientific and Technological Research Council of Türkiye (TÜBİTAK 1007). To obtain cutting material, selected tea plants were subjected to severe pruning in March. Leaf-bud cuttings were collected from the shoots that developed after pruning on 8 August 2024. Seed material was obtained from mature fruits, and seeds were sown on 12 November 2024.

For each genotype, 48 leaf-bud cuttings and 48 seeds were initially used, giving a total of 96 propagation units per genotype. In total, 768 propagation units (8 genotypes × 96 units) were initially established. All nursery plants were grown under the same greenhouse conditions and subjected to the same cultural practices throughout the experiment. Propagation success was calculated based on the initial number of cuttings planted or seeds sown. Successfully established plants were subsequently transplanted into individual pots and arranged into three replicates for growth and quality assessments. The mean value of each replicate was used as the experimental unit for statistical analysis.

### 2.2. Preparation and Planting of Cuttings

**Figure 1:**
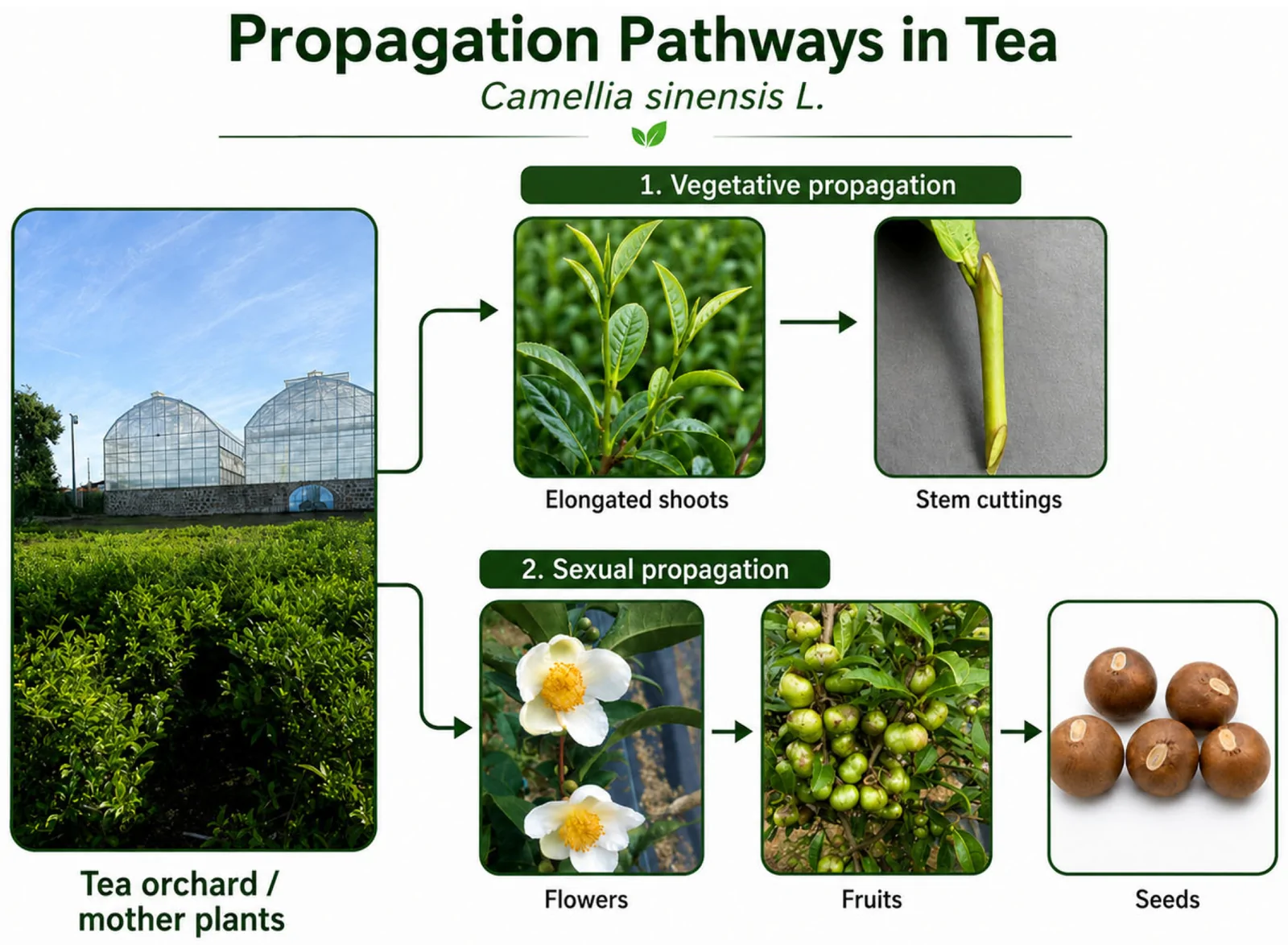
Schematic representation of propagation material collection from tea (*Camellia sinensis* L.) mother plants.

For cutting propagation, shoot development was stimulated by severe pruning of the selected genotypes and cultivars. After pruning, single-node leaf-bud cuttings were prepared from healthy, mature, and semi-hardwood shoots. The use of single-node leaf-bud cuttings has been reported as one of the most common and successful methods for clonal propagation in tea plants [20,21]. The cuttings were prepared as single-node segments, approximately 3–4 cm in length, and the cut was made at a 45° angle just above the axillary bud at the leaf node (Figure 1). This method is considered one of the standard propagation practices for promoting rooting in tea plants [12,22].

To promote rooting, the basal ends of cuttings were treated with 4000 mg L^−1^ indole-3-butyric acid (IBA; Merck KGaA, Darmstadt, Germany) using a quick-dip method. The IBA solution was prepared by first dissolving IBA in 96% ethanol and then bringing the solution to the final volume with distilled water. The basal portion of each cutting was immersed in the IBA solution for 10 s and then immediately planted in the rooting medium consisting of a peat/vermiculite mixture (3:1, *v*/*v*) with a pH of 5.5. After planting, regular irrigation was applied, and greenhouse temperature and humidity were maintained under controlled conditions. During the rooting and growing periods, greenhouse temperature varied seasonally from approximately 10–12 °C in winter to about 26 °C in late summer and increased to approximately 19 °C in spring, while relative humidity generally remained between 68% and 78%. During the rooting period, the nursery plants were regularly monitored for diseases and pests.

### 2.3. Preparation and Sowing of Seeds

In seed propagation, healthy and fully filled seeds obtained from mature tea fruits were selected. Before sowing, the seeds were physically examined and soaked in water for 24 h to promote germination. The selected seeds were sown in plug trays containing a peat/vermiculite mixture (3:1, *v*/*v*), the same growing medium used for cutting propagation. After sowing, regular irrigation was applied, and greenhouse temperature and relative humidity were maintained under controlled conditions. Germination and root development were monitored regularly throughout the nursery plant growth period.

### 2.4. Assessment of Nursery Plant Growth and Quality Parameters

In the study, propagation success (%), root development level (1–5), shoot quality (1–5), stem diameter (mm), shoot length (cm), root weight (g), and root length (cm) were evaluated. Propagation success (%) was calculated separately for each propagation method. For seed-propagated plants, it was calculated as the number of germinated and successfully established plants divided by the total number of seeds sown. For cutting-propagated plants, it was calculated as the number of successfully established cuttings showing shoot development divided by the total number of cuttings planted. Values were expressed as percentages. Shoot quality was visually assessed using a 1–5 ordinal scale based on overall shoot vigor and development. Stem diameter was measured using a KMP 150 digital caliper (0–150 mm measuring range, 0.01 mm resolution), while shoot length and root length were measured with a ruler. Root weight was determined by weighing the roots using a Pioneer™ precision balance (OHAUS Corporation, Parsippany, NJ, USA) with an accuracy of 0.001 g [12].

**Figure 2:**
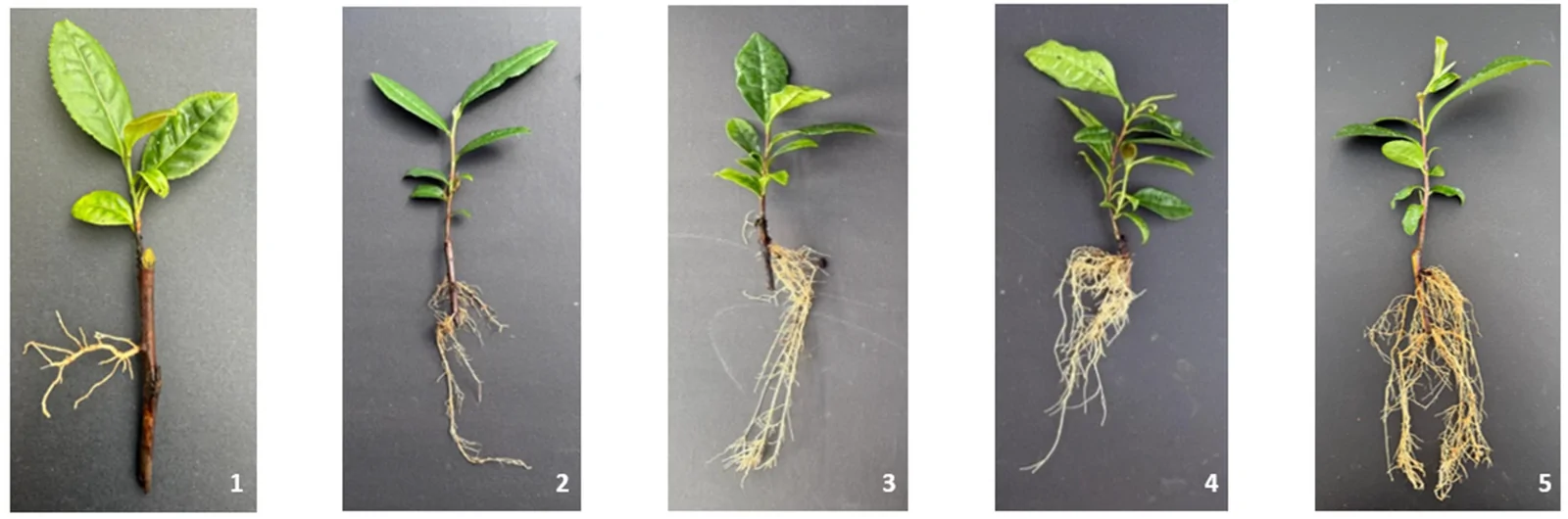
Root development scale (TeaRDS) developed in the present study for cutting-propagated tea nursery plants (1: very poor root development; 2: poor root development; 3: moderate root development; 4: good root development; 5: very good root development).

**Figure 3:**
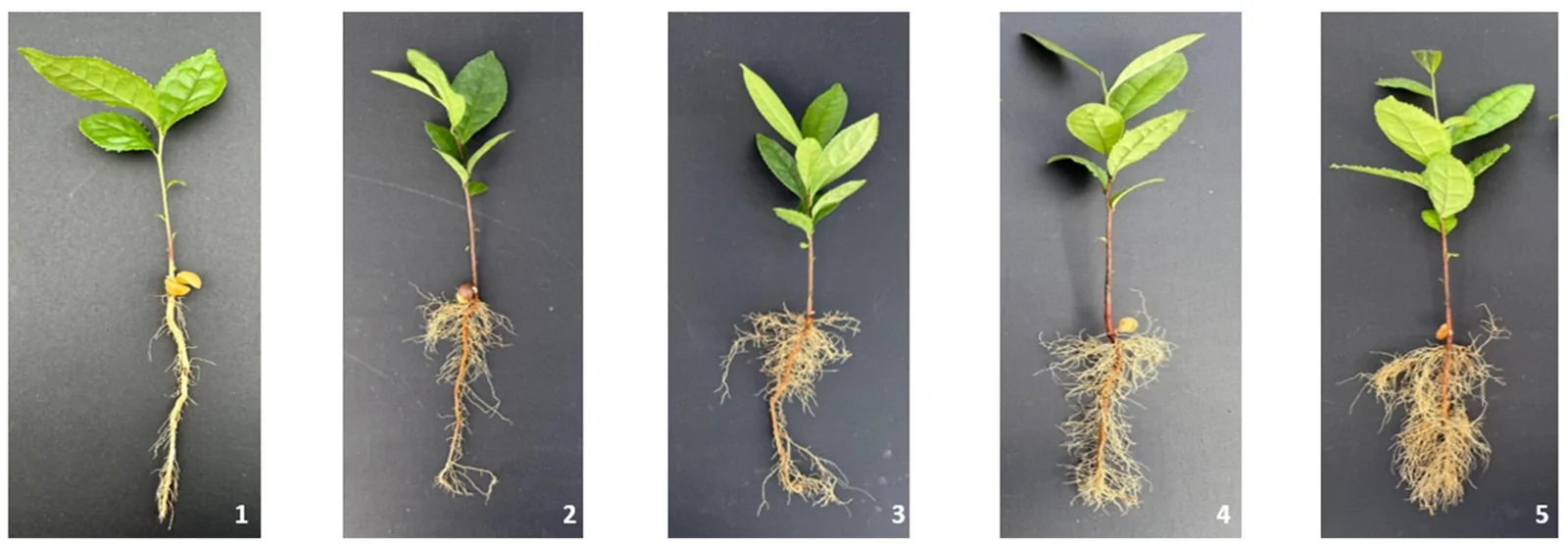
Root development scale (TeaRDS) developed in the present study for seed-propagated tea nursery plants (1: very poor root development; 2: poor root development; 3: moderate root development; 4: good root development; 5: very good root development).

Root development was assessed using separate five-level Tea Root Development Scales (TeaRDS) developed in the present study for cutting- and seed-derived tea nursery plants (Figure 2 and Figure 3), reflecting their distinct root system architectures. For cutting-derived plants, scores 1–5 represented: very limited adventitious rooting with sparse roots (1); limited rooting with few roots and low branching (2); moderate rooting with intermediate branching and fine-root development (3); well-developed rooting with extensive branching and fine roots (4); and a dense, highly branched root system with extensive fine-root development (5). For seed-derived plants, scores 1–5 represented: a weak taproot with very limited lateral roots (1); a developed taproot with limited lateral roots and branching (2); a moderately developed taproot with intermediate lateral root formation and branching (3); a well-developed taproot and lateral root system with good branching and root density (4); and a strong taproot with abundant lateral roots and extensive branching (5). TeaRDS was used as an ordinal visual assessment framework rather than as a substitute for direct quantitative root measurements.

### 2.5. Statistical Analysis

For statistical analyses, all eight plant materials were treated collectively as the genotype factor (G). The effects of genotype (G), propagation method (M), and the genotype × propagation method (G × M) interaction were evaluated. A two-way analysis of variance (ANOVA) was performed for stem diameter (SD), shoot length (SL), root length (RL), and root weight (RW), with effect sizes expressed as partial eta-squared (ηp^2^). When significant effects were detected, pairwise comparisons were performed using Tukey-adjusted multiple comparisons at *p* < 0.05. The Scheirer–Ray–Hare test was applied to shoot quality (SQ) and root development (RD)—which are ordinal variables—and effect sizes were calculated using epsilon-squared (εr^2^). Multivariate relationships were assessed using PCA, PCoA based on Gower distance, Pearson correlation analysis, and a Euclidean distance matrix derived from standardized data. Results were visualized using box plots, interaction plots, and heatmaps. All statistical analyses and visualizations were conducted using R software (version 4.6.1); the threshold for statistical significance was set at *p* < 0.05, and significant results were reported at the *p* < 0.05, *p* < 0.01, and *p* < 0.001 levels.

## 3. Results

### 3.1. Observations on Germination, Callus Formation, and Rooting Processes

**Figure 4:**
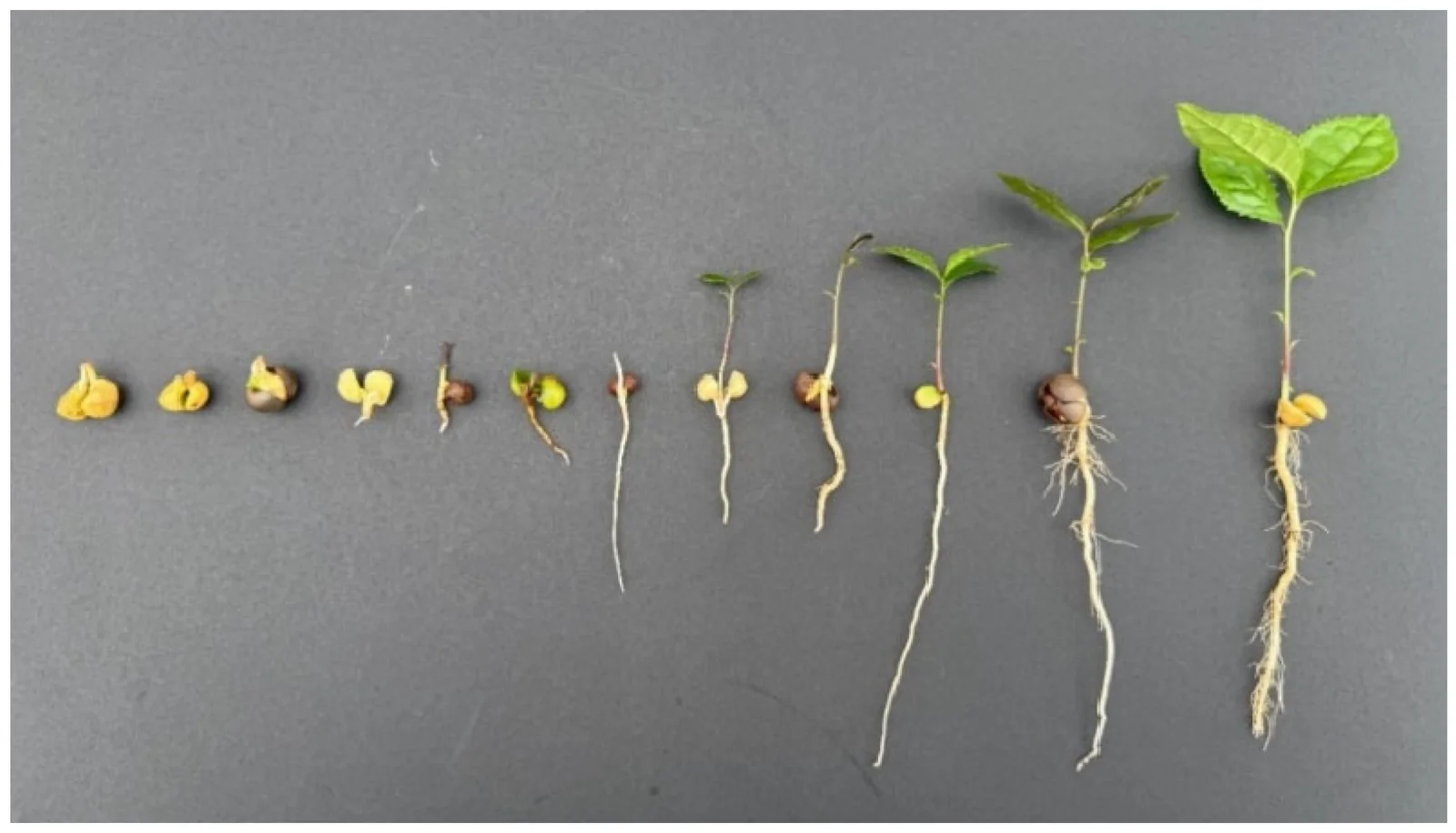
Sequential stages of tea seed germination and seedling development.

In seed-propagated tea plants, the taproot first developed prominently during the rooting process and showed vertical growth. Subsequently, lateral roots formed around the taproot. Although plant growth was initially slow, faster growth was observed after the formation of lateral roots. The germination stages of tea seeds are shown in Figure 4. Approximately 90 days passed from the first stage to the final stage shown in the figure.

**Figure 5:**
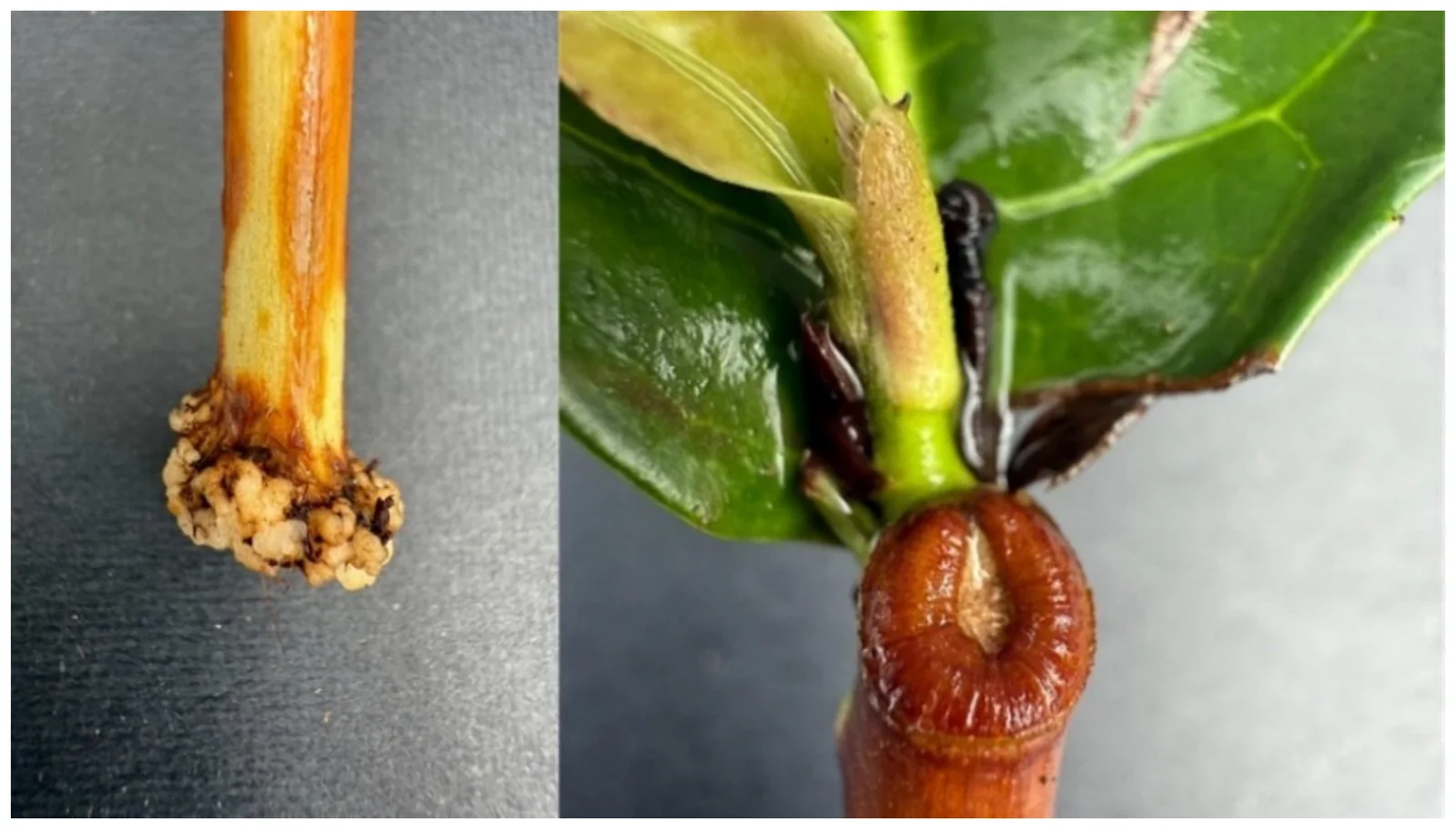
Callus formation at the basal wound surface of tea cuttings.

In cutting propagation, callus formation was observed on the wound surface approximately four weeks after planting (Figure 5). Following callus formation, the first root initials were observed on the callus tissue after 3–4 weeks, and visible fibrous root formation was detected within 6–8 weeks.

**Figure 6:**
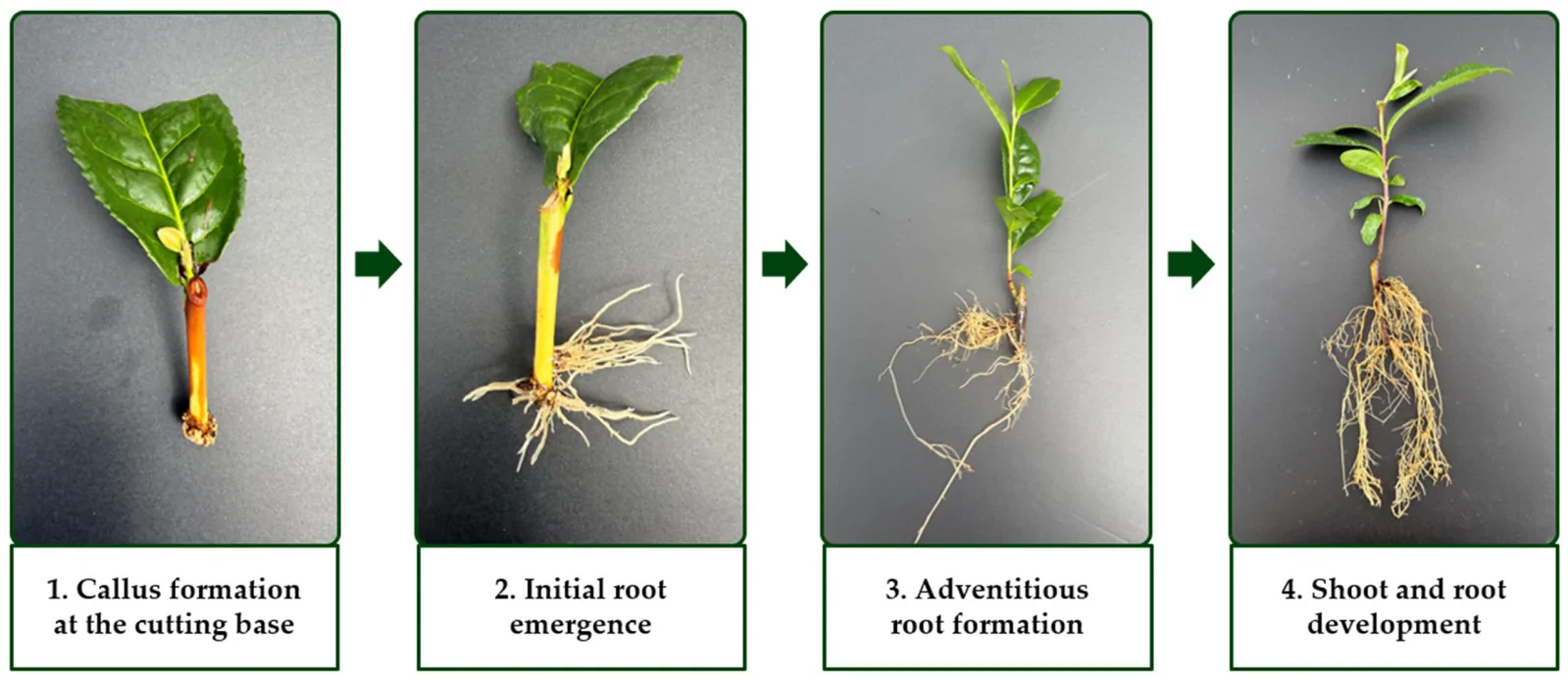
Sequential stages of callus formation, adventitious rooting, and shoot development in tea cuttings.

Visual observations showed fine adventitious root development, mainly concentrated at the basal region of the cuttings (Figure 6). Although the roots were located close to the soil surface, they developed rapidly.

### 3.2. Effects of Genotype and Propagation Method on Growth and Nursery Plant Quality

The effects of genotype (G), propagation method (M), and the G × M interaction on growth and quality parameters were evaluated, and the results are presented in Table 1, which shows that genotype, propagation method, and the G × M interaction affected the examined parameters to varying degrees.

The propagation method had a highly significant effect on stem diameter (SD), shoot length (SL), and root weight (RW) (*p* < 0.001), and a significant effect on shoot quality (SQ) (*p* = 0.031). In particular, the high effect sizes calculated for SD, SL, and RW (ηp^2^ = 0.300–0.633) indicated a substantial influence of the propagation method on these traits. The genotype effect was significant for root development (RD) (*p* = 0.008) and highly significant for shoot length (SL) and root weight (RW) (*p* < 0.001). In contrast, genotype and propagation method effects on root length (RL) were not statistically significant. The G × M interaction was significant for stem diameter (SD), shoot quality (SQ), and root weight (RW) (*p* < 0.05), whereas it was not significant for the other parameters.

### 3.3. Propagation Success (%)

**Figure 7:**
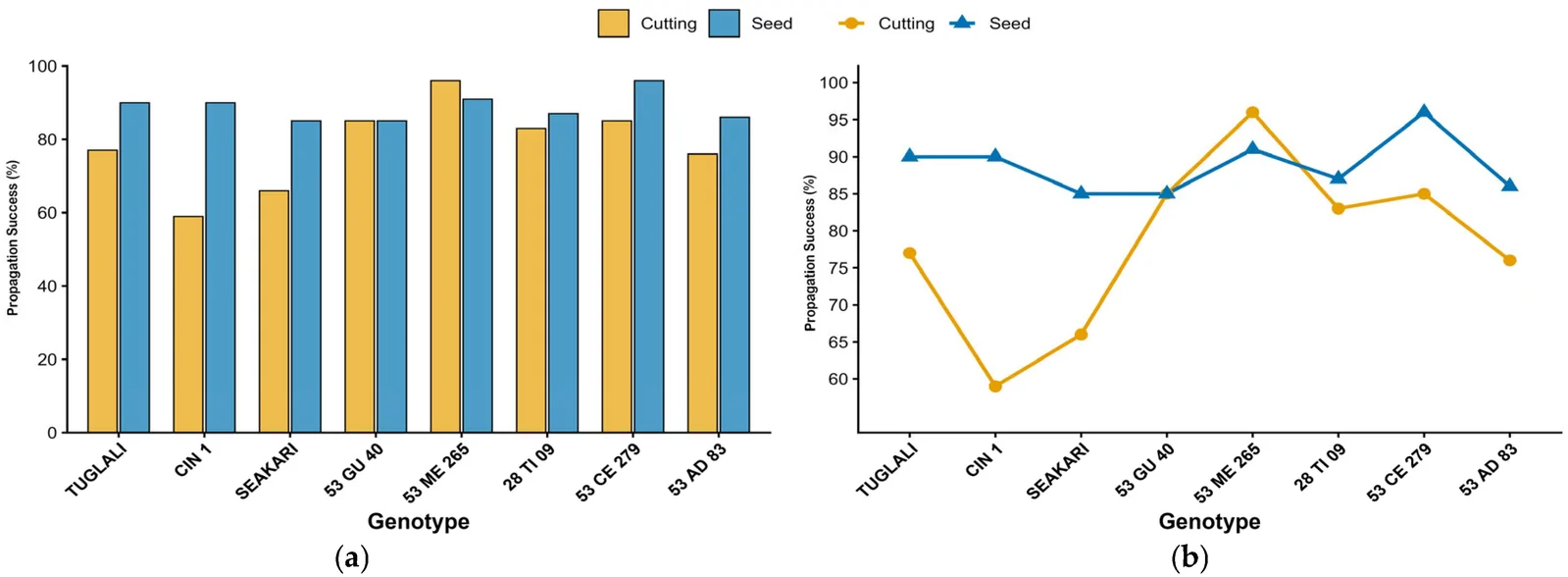
Comparison of propagation success in different tea genotypes propagated by cuttings and seeds. (**a**) Propagation success (%) by genotype and propagation method. (**b**) Genotype-dependent variation profile of propagation success in cutting- and seed-derived nursery plants.

An examination of propagation success in nursery plants produced by cuttings and seeds across different tea genotypes showed that, overall, seed propagation showed numerically higher propagation success than cutting propagation. However, propagation success varied markedly among genotypes. Notably, the 53 ME 265 and 53 GU 40 genotypes exhibited relatively high propagation success when propagated by cuttings, whereas CIN-1 showed lower success under cutting propagation compared with the other genotypes. The line graph further indicated genotype-dependent differences in the response to propagation method, with some genotypes showing similar propagation success under both cutting and seed propagation (Figure 7).

### 3.4. Root Development (1–5)

**Figure 8:**
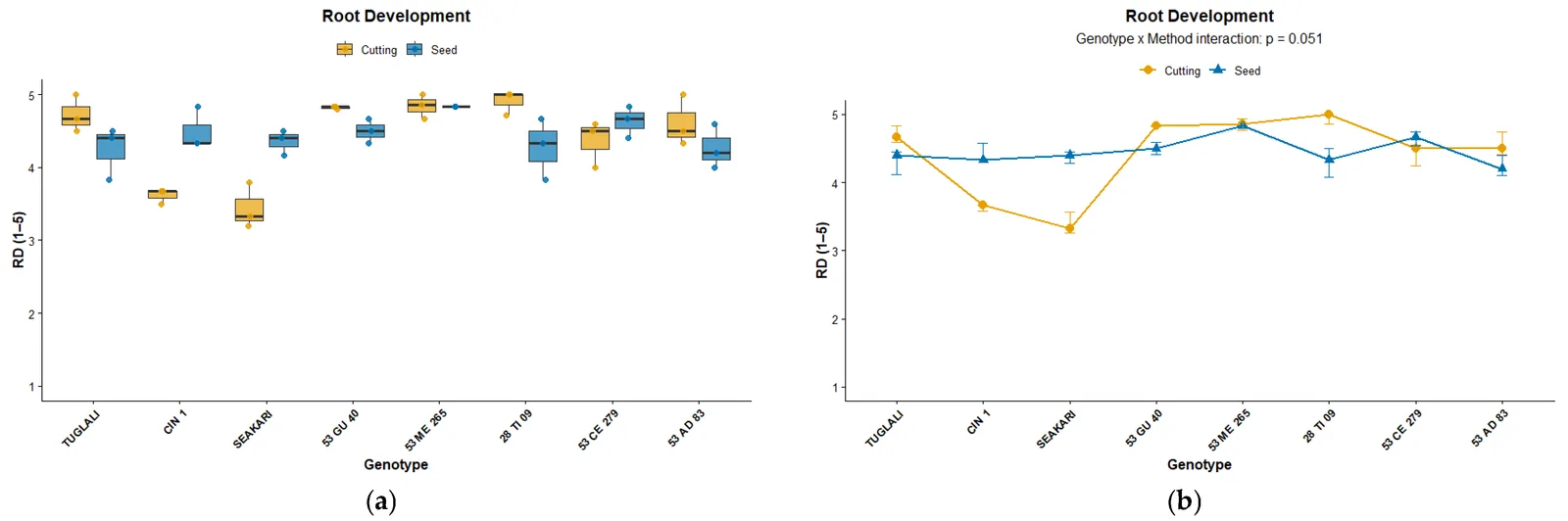
Comparison of root development level in different tea genotypes propagated by cuttings and seeds. (**a**) Statistical differences in root development level among genotypes. (**b**) Genotype-dependent variation profile of root development level according to propagation methods.

Root development was evaluated using a 1–5 rating scale. According to the Scheirer–Ray–Hare test, the genotype effect was statistically significant (*p* = 0.008), whereas the effect of propagation method was not significant (*p* = 0.437). These results indicated that variation in root development level was generally more strongly associated with genotypic differences than with propagation method. The graphical pattern suggested some genotype-dependent variation in the response to propagation method (Figure 8).

### 3.5. Shoot Quality (1–5)

**Figure 9:**
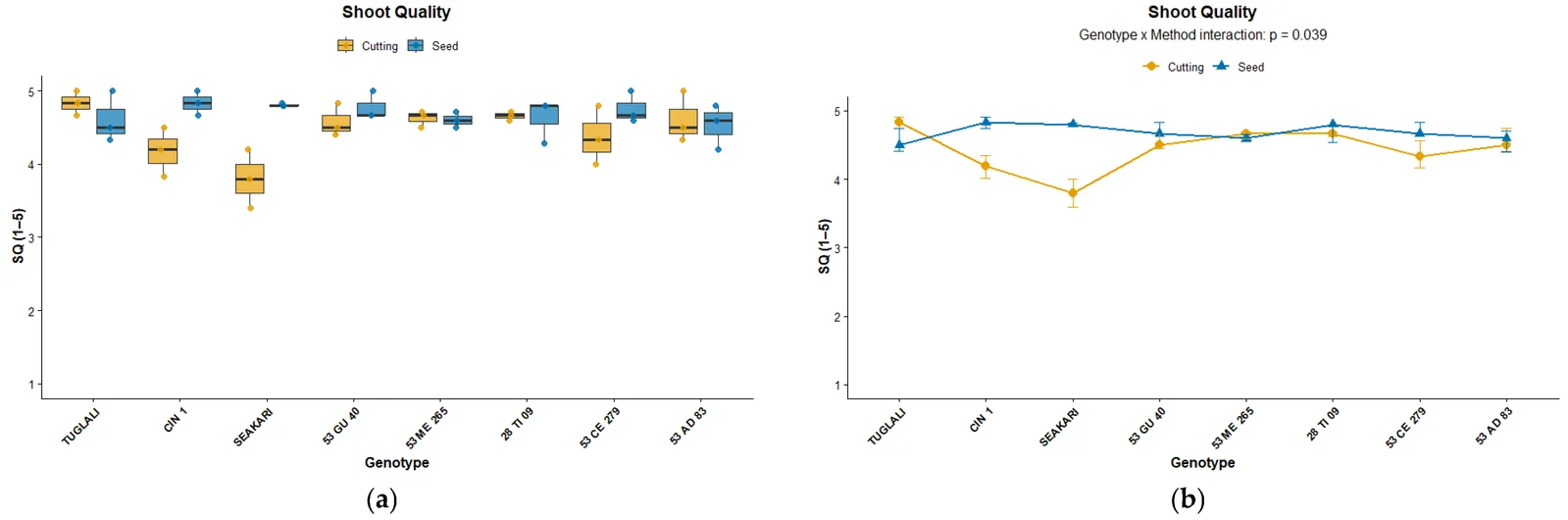
Comparison of shoot quality values in different tea genotypes propagated by cuttings and seeds. (**a**) Distribution of shoot quality scores according to genotype and propagation method. (**b**) Genotype-dependent variation profile of shoot quality according to propagation methods.

Shoot quality was evaluated using a 1–5 rating scale. According to the Scheirer–Ray–Hare test, the effect of propagation method on shoot quality was significant (*p* = 0.031), whereas the genotype effect was not significant (*p* = 0.933). The genotype × propagation method interaction was also statistically significant for shoot quality (*p* = 0.039). In addition, shoot quality values showed different distribution patterns among genotype × propagation method combinations. The high shoot quality scores observed in some cutting-propagated combinations indicated that cutting-derived nursery plants could achieve good-quality development in suitable genotypes (Figure 9).

### 3.6. Stem Diameter (mm)

**Figure 10:**
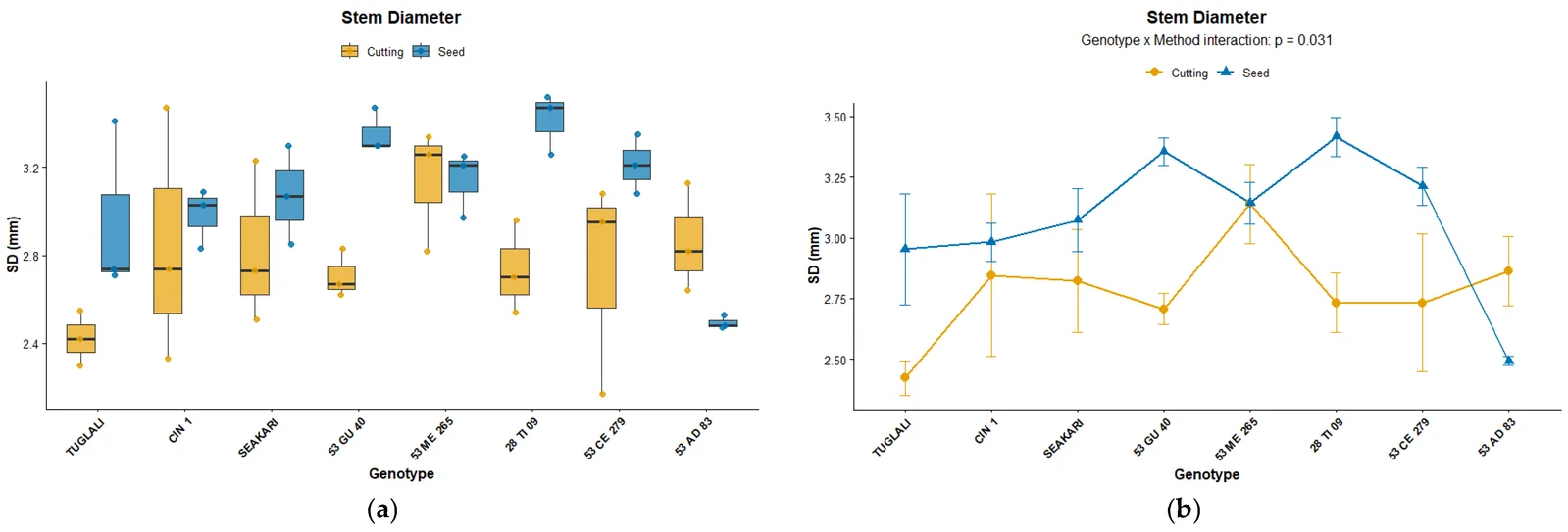
Comparison of stem diameter in different tea genotypes propagated by cuttings and seeds. (**a**) Stem diameter values according to genotypes. (**b**) Genotype-dependent variation profile of stem diameter according to propagation methods.

The propagation method had a highly significant effect on stem diameter (*p* < 0.001). The G × M interaction also had a significant effect on this parameter (*p* = 0.031). In contrast, the genotype effect was close to the significance threshold but was not statistically significant (*p* = 0.056). In general, seed-derived nursery plants exhibited greater stem diameter values. However, the magnitude of this difference varied among genotypes, and some cutting- and seed-derived combinations showed similar values (Figure 10).

### 3.7. Shoot Length (cm)

**Figure 11:**
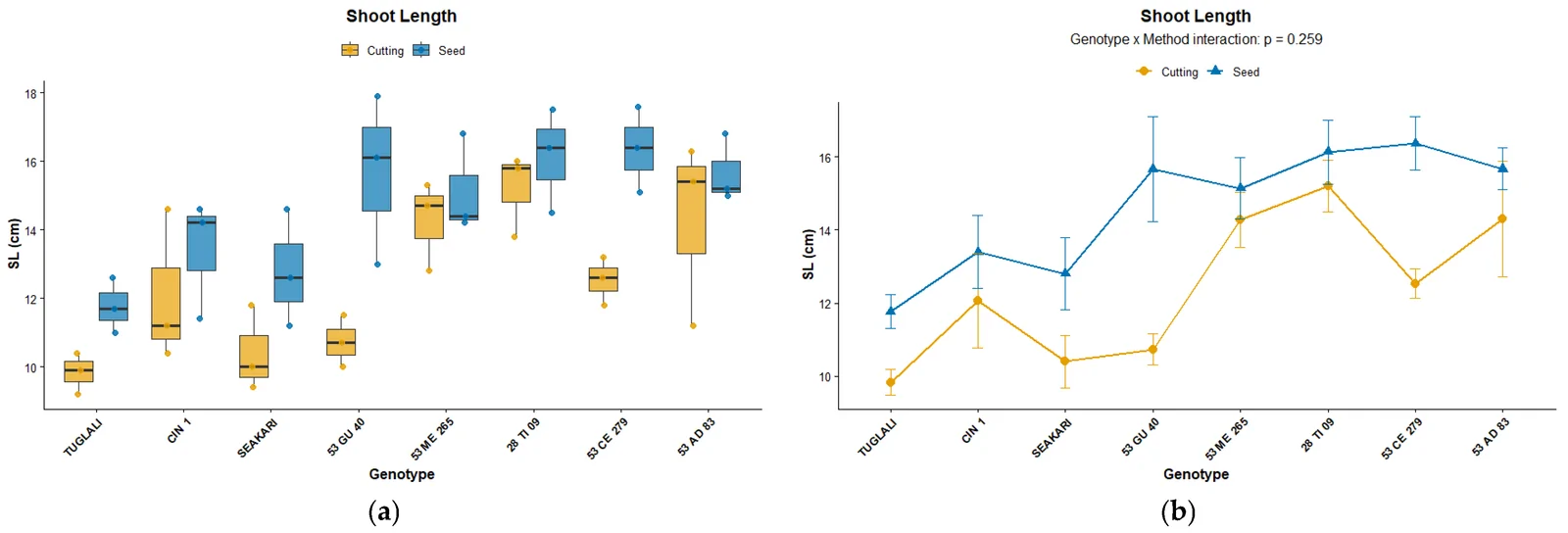
Comparison of shoot length in different tea genotypes propagated by cuttings and seeds. (**a**) Shoot length values according to genotypes. (**b**) Genotype-dependent variation profile of shoot length according to propagation methods.

Both genotype and propagation method had significant effects on shoot length (*p* < 0.001). In contrast, the G × M interaction was not significant for shoot length (*p* = 0.259). In general, seed-propagated nursery plants exhibited greater shoot lengths than cutting-derived plants. However, in some genotypes, such as 28 TI 09 and 53 ME 265, the difference between cutting and seed treatments was more limited (Figure 11).

### 3.8. Root Weight (g)

**Figure 12:**
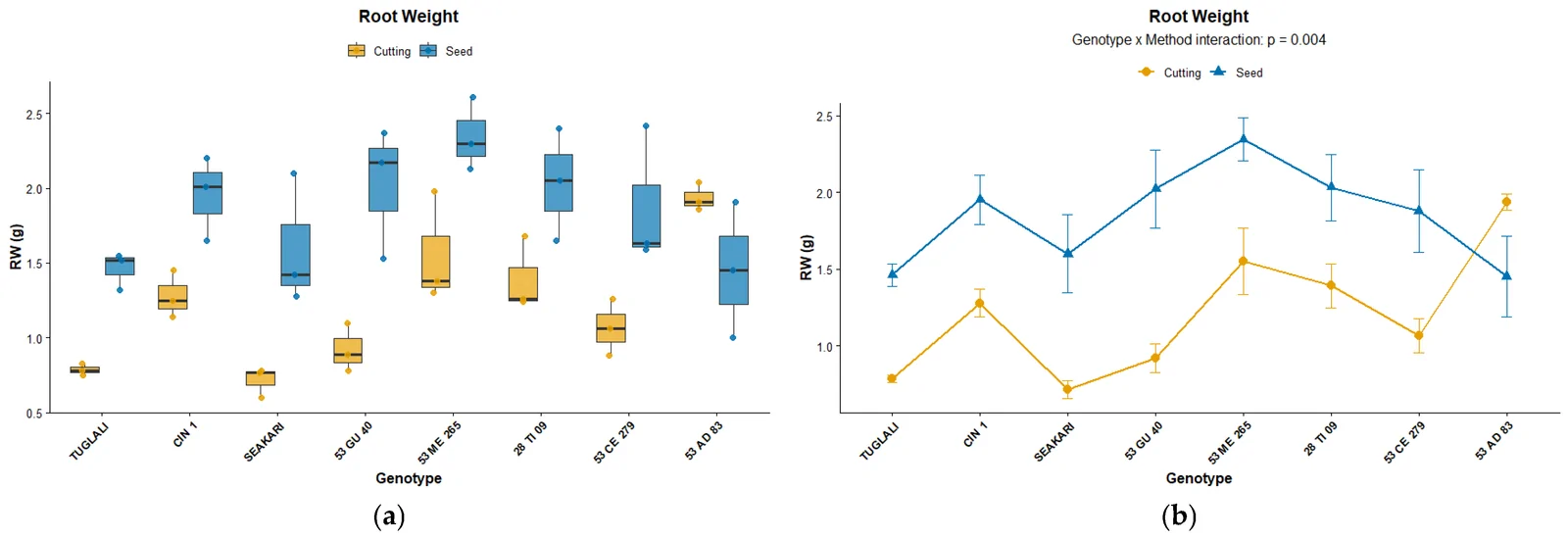
Comparison of root weight in different tea genotypes propagated by cuttings and seeds. (**a**) Root weight values according to genotypes. (**b**) Genotype-dependent variation profile of root weight according to propagation methods.

Genotype, propagation method, and the G × M interaction all had significant effects on root weight. Both the genotype effect and the propagation method effect were significant at *p* < 0.001, while the G × M interaction was significant at *p* = 0.004. The high effect size of the propagation method for this parameter (ηp^2^ = 0.633) indicated that root weight was strongly influenced by the propagation method. In general, root weight values were greater in seed-derived nursery plants; however, the magnitude of this difference varied among genotypes, and some cutting-derived nursery plants showed values close to those of seed-derived plants (Figure 12).

### 3.9. Root Length (cm)

**Figure 13:**
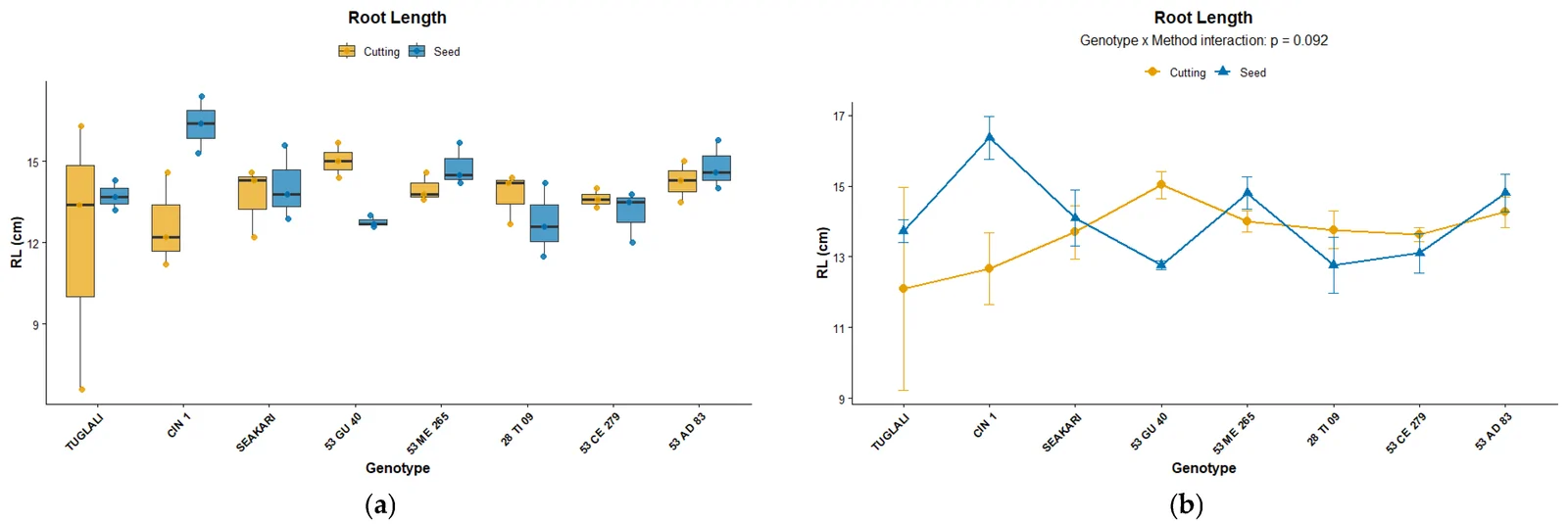
Comparison of root length in different tea genotypes propagated by cuttings and seeds. (**a**) Root length values according to genotypes. (**b**) Genotype-dependent variation profile of root length according to propagation methods.

For root length, the effects of genotype (*p* = 0.499), propagation method (*p* = 0.374), and the G × M interaction (*p* = 0.092) were not statistically significant. Thus, no significant effects of genotype, propagation method, or their interaction on root length were detected. Although some differences were observed among genotype × propagation method combinations for root length, these differences did not reach statistical significance (Figure 13).

### 3.10. Multivariate Analyses and Relationships Among Parameters

The growth and quality characteristics of nursery plants propagated via cuttings and seeds were evaluated using complementary multivariate analysis methods. When findings from PCoA, Euclidean distance matrix, PCA, and correlation analyses were assessed collectively, it was determined that certain genotypes propagated by cuttings exhibited growth and development profiles similar to those of seed-propagated nursery plants. Notably, 53 ME 265 and 53 AD 83 showed the closest cutting–seed profiles, while 53 CE 279 and 28 TI 09 also showed relatively close positioning in the multivariate analyses.

**Figure 14:**
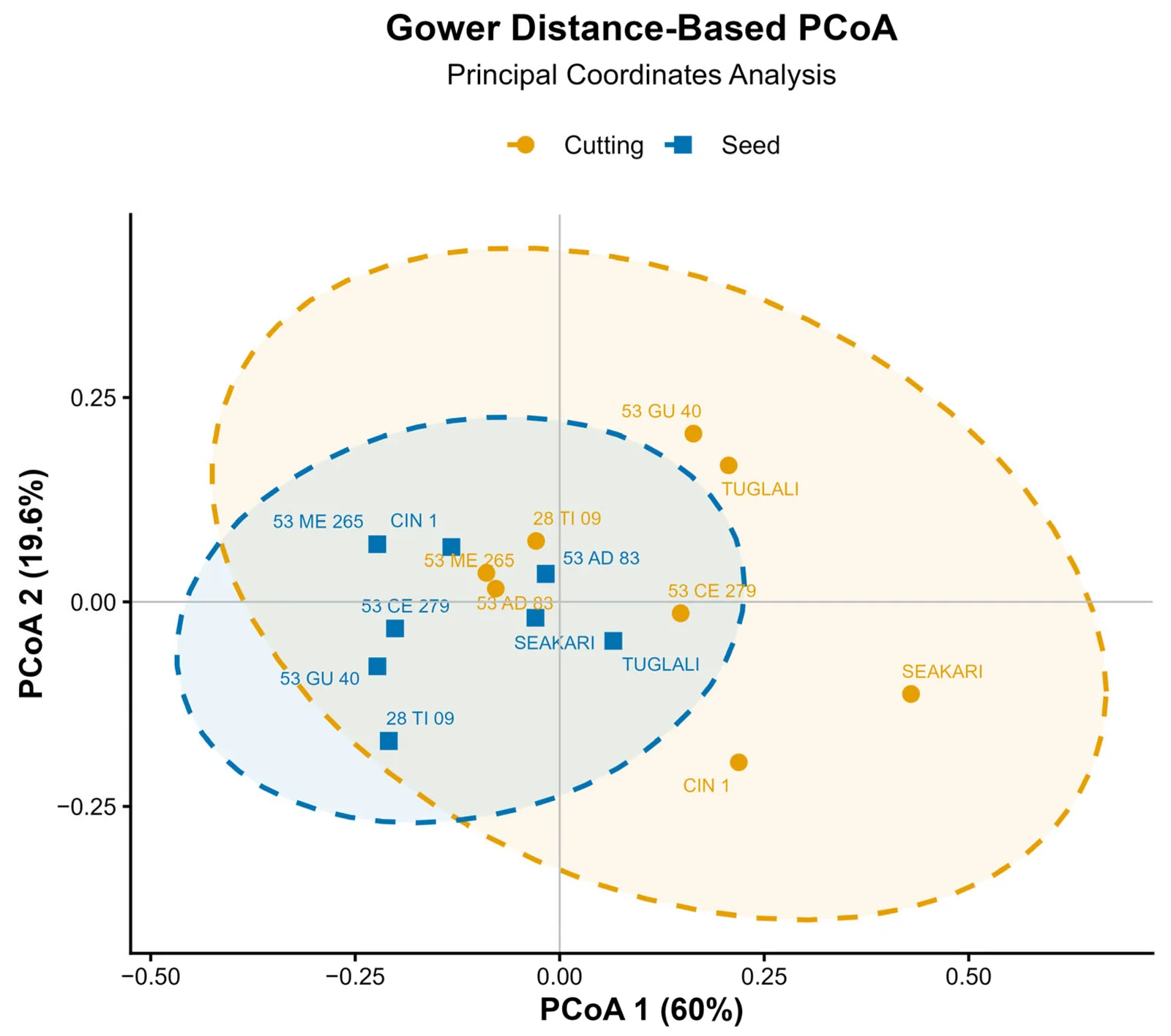
Principal Coordinates Analysis (PCoA) based on Gower distance. The distribution of genotypes according to propagation method (cuttings and seeds) is shown. The first two axes explained a substantial proportion of the total variation (PCoA1: 60%; PCoA2: 19.6%), and partial separation as well as clear overlap were observed between the samples propagated by cuttings and seeds. The ellipses represent the 95% confidence interval.

In the PCoA based on Gower distance, the first axis (PCoA1) explained 60% of the total variance, while the second axis (PCoA2) explained 19.6%; together, the two axes accounted for 79.6% of the total variation. Although a partial separation was observed between the cutting- and seed-propagated samples in the PCoA biplot, a considerable overlap remained between the groups. This indicates that propagation method contributed to multivariate differences in nursery traits, but its effect was not uniform across genotypes. Cutting combinations for genotypes 53 AD 83, 53 ME 265, 53 CE 279, and 28 TI 09 were found to be positioned close to certain seed combinations (Figure 14).

**Figure 15:**
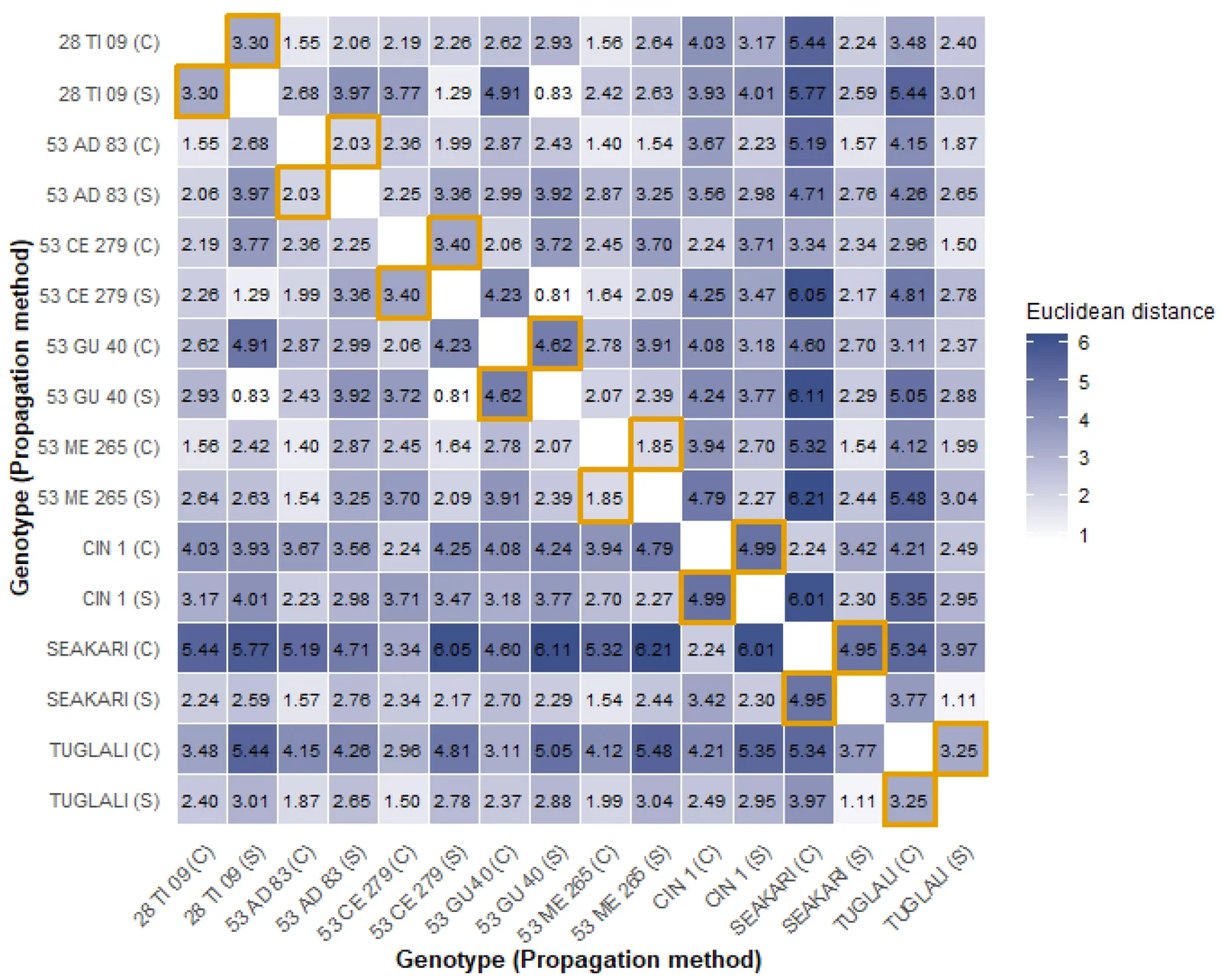
Euclidean distance matrix heatmap based on the normalized growth parameters of nursery plants propagated by cuttings (C) and seeds (S) in tea genotypes. Orange boxes highlight the Euclidean distance values between cutting (C) and seed (S) propagation methods within each genotype.

The Euclidean distance heatmap revealed clear multivariate differences among genotype × propagation method combinations. Euclidean distances between cutting (C) and seed (S) applications of the same genotype varied depending on the genotype. The lowest distance values were determined in genotypes 53 ME 265 (d = 1.85) and 53 AD 83 (d = 2.03), and it was observed that the growth and development profiles of the two propagation methods were close to each other in these genotypes. In contrast, higher distance values were determined in genotypes CIN-1 (d = 4.99), SEAKARI (d = 4.95), and 53 GU 40 (d = 4.62) (Figure 15).

Pearson correlation analysis revealed relationships among the evaluated parameters. Root weight (RW) and shoot length (SL) (r = 0.63), shoot quality (SQ) and root development (RD) (r = 0.59), and root weight (RW) and stem diameter (SD) (r = 0.55) showed positive correlations. In contrast, root length (RL) showed weaker correlations with other parameters and showed a relatively independent pattern.

**Figure 16:**
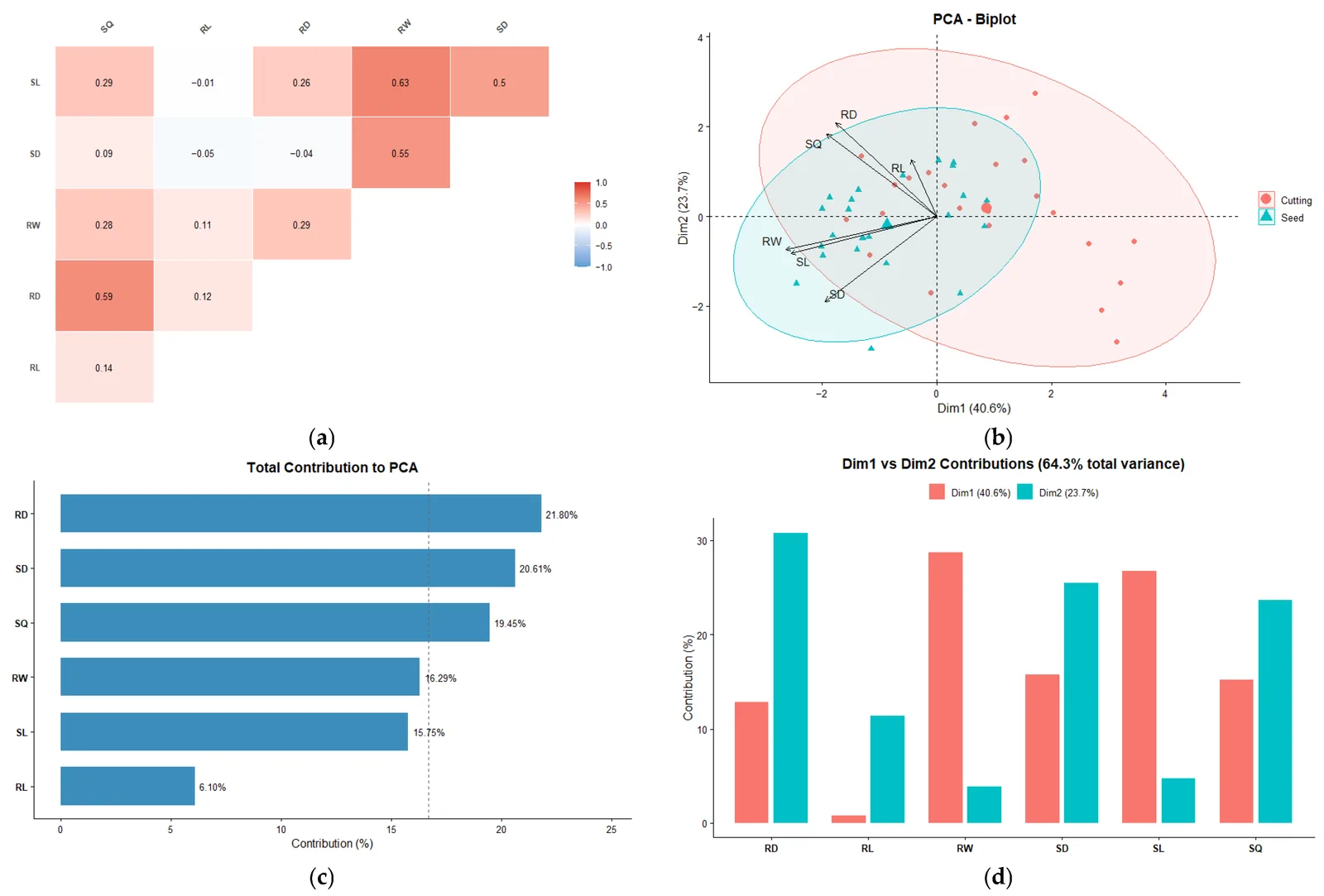
Correlation and principal component analysis of growth and quality characteristics of tea nursery plants propagated by cuttings and seeds. (**a**) Pearson correlation matrix between parameters. (**b**) PCA biplot according to the first two principal components; vectors show the loading directions of the variables. (**c**) Mean contributions of the variables to the first two PCA dimensions. (**d**) Contributions of the variables to Dim1 and Dim2.

Principal component analysis (PCA) explained 64.3% of the total variance under the first two components (Dim1: 40.6%; Dim2: 23.7%). The similar loadings of root weight (RW), shoot length (SL), and stem diameter (SD) in the PCA biplot indicated that these variables collectively represent overall growth performance. Conversely, the similar loadings of SQ and RD supported the relationship between shoot quality and root development. When the distribution of the samples was examined, it was observed that there was no complete separation between nursery plants propagated by cuttings and seeds, and some cutting combinations were located close to seed combinations. In terms of average contribution to the first two PCA dimensions, RD (21.80%), SD (20.61%) and SQ (19.45%) stood out, while RL showed the lowest contribution (6.10%) (Figure 16).

## 4. Discussion

The contrasting performance of seed- and cutting-derived tea nursery plants may reflect fundamental differences in early root establishment and resource allocation between the two propagation systems. Overall, seed-propagated nursery plants showed greater shoot length, stem diameter, and root weight, consistent with the significant effects of propagation method on these traits. Propagation success was also numerically higher for seed propagation in several genotypes. This may be related to the initial nutrient reserves of seeds, their capacity to form a primary root system, and the early development advantage in generative propagation. The early, prominent development of the taproot in seed-propagated plants, followed by lateral root formation, indicates a vertically oriented and deepening root system at early stages. In contrast, the rooting process in nursery plants propagated by cuttings progressed through callus formation and adventitious root development [15]. Auxin treatments such as IBA have been reported to promote adventitious root formation in tea cuttings, with auxin-related signaling and gene-expression changes playing important roles during root development [13,14]. Therefore, callus formation and adventitious root development observed in this study are consistent with studies reporting rooting processes controlled by hormonal regulation [23,24,25]. The promotive role of IBA in adventitious rooting of tea cuttings is already well established. The present study extends this knowledge by showing that, even under a standardized IBA treatment and common greenhouse conditions, nursery performance remains strongly genotype-dependent.

The genotype-dependent response to cutting propagation indicates that rooting capacity is strongly influenced by the intrinsic characteristics of the plant material. These findings are consistent with studies reporting that the success of tea propagation by cuttings is affected by factors such as genotype, cutting time, physiological maturity, and rooting conditions [12,21,26,27]. The differential response of tea genotypes to cutting propagation may be associated with genotype-specific differences in the physiological capacity for adventitious root formation, including variations in endogenous hormone balance, carbohydrate availability, tissue maturity, and subsequent root initiation and development.

The close distribution of some cutting and seed combinations in terms of shoot quality and root development supports the idea that nursery plants propagated by cuttings can show comparable characteristics to nursery plants propagated by seed in terms of root system development. In particular, the absence of a significant propagation-method effect on root development suggests, without establishing formal equivalence, that cuttings from suitable genotypes can achieve adequate root development. This variability observed in propagation reveals the importance of genetic variation and genotype-specific rooting capacity. This is consistent with studies previously conducted on different plant species and tea [27,28,29]. Similarly, differences in growth and root development between seedlings of seed and vegetative origin have also been shown to vary depending on species and genotype, as supported by studies conducted on various woody species such as cedar, Chinese fir, black locust, and chestnut [30,31,32,33]. One of the methodological contributions of the study is the five-level Tea Root Development Scales (TeaRDS), developed to evaluate the root system of tea nursery plants in a standard visual framework. The significant genotypic difference in scale-assessed root development (H(7) = 18.968; *p* = 0.008; εr^2^ = 0.299) indicates that this approach can distinguish genotype-dependent variation in root development. However, RD was only weakly correlated with directly measured root weight (r = 0.29), indicating that the visual scale does not substitute for gravimetric measurement. In contrast, the positive relationship between shoot quality and root development at r = 0.59 indicates that visual root development is a characteristic related to overall shoot quality. The visual-ordinal approach of the TeaRDS shows conceptual similarity to root and nursery plant quality assessment systems used in different plant groups. The Dickson Quality Index aims to holistically assess nursery plant quality by combining root and shoot biomass, root collar diameter, and plant height [34]. Similarly, approaches have been developed to evaluate root development potential or root system architecture across different species; for instance, the root architecture of Chinese fir (*Cunninghamia lanceolata*) seedlings originating from seeds, cuttings, and tissue culture has been comparatively evaluated [31]. TeaRDS is significant for offering a simple and practical photographic reference framework specifically for tea—that condenses the overall development of the root system (such as density, branching, and fine root formation) into a single score. However, for the scale to be used as an independent measurement tool, inter-rater reliability and validity analyses with different root characteristics need to be conducted in subsequent studies. These patterns indicate that propagation method influences overall nursery growth, although the magnitude of this response is genotype-dependent. However, some genotypes showing cutting-derived values quite close to seed-derived plants support the idea that the response to propagation method varies with genotype [27,35,36,37].

The absence of significant genotype, propagation-method, and interaction effects on root length suggests that this trait was less responsive to these factors than the other evaluated growth traits. The weak correlation of root length with other parameters also supports this result. This suggests that root length alone may not be sufficient to explain root system performance; it should be evaluated together with parameters such as root weight, root development level, and shoot quality. These results are consistent with studies in the literature in which root development and rooting performance were evaluated using multiple complementary traits [12,26,38,39,40]. The multivariate analyses further supported the genotype-dependent nature of the propagation response. The overlap between cutting- and seed-derived groups indicates that propagation method did not produce a uniform developmental pattern across all genotypes.

Euclidean distance analysis also showed that the degree of similarity between cutting- and seed-derived plants varied markedly among genotypes. This supports the view that cutting propagation can produce developmental profiles comparable to seed propagation in selected genotypes, but not uniformly across all plant materials. PCA and correlation analyses showed that root weight, shoot length, stem diameter, and root development parameters are key variables that collectively represent overall growth performance. Positive relationships between these parameters reveal that nursery plants with strong root development can also show higher shoot development and stem diameter. In contrast, the more independent position of root length compared to other parameters indicates that root quality in tea nursery plants should be evaluated not only by root length but also by root weight and root development level. Some limitations of the study should also be considered. The limited sample size in genotype × method combinations may have reduced statistical power, especially in effects close to the significance level. For example, the genotype effect being *p* = 0.056 in SD and the G × M interaction being *p* = 0.051 in RD necessitate a cautious interpretation of these results. In addition, the root development scale has not yet been validated across different evaluators and independent materials, which limits its generalizability. The single-greenhouse setting and early developmental stage examined in this study should also be considered when generalizing to different environments and age groups. In addition, nursery-stage differences should not be interpreted as direct predictors of field survival, long-term yield, or stress resilience after transplanting. Long-term field trials are needed to determine whether these differences persist under field conditions. Overall, this study revealed that the propagation method is an important factor in the growth performance of tea nursery plants, but this effect varies depending on the genotype. The convergence of the multivariate profiles of nursery plants from cuttings and seed-derived plants, particularly in 53 ME 265 and 53 AD 83, supports this result. Although seed propagation resulted in greater overall shoot length, stem diameter, and root weight, cutting propagation produced comparable developmental profiles in selected genotypes. These findings highlight the importance of genotype selection and indicate that propagation by cuttings may represent a viable approach to produce genetically homogeneous clonal tea nursery plants, particularly in plantation renewal programs.

## 5. Conclusions

This study comparatively evaluated the effects of seed and cutting propagation methods on growth, root development, and shoot quality in tea plants. The findings revealed that the propagation method had significant effects, particularly on shoot development, stem diameter, and root weight, while the magnitude of these effects varied among genotypes. In some genotypes, cutting-derived nursery plants showed developmental profiles like those of seed-derived plants, particularly in traits related to root system development.

Multivariate analyses supported this genotype-specific response, showing that the degree of similarity between cutting- and seed-derived nursery plants differed among genotypes. No significant effect of propagation method on root development was detected, although the present design does not allow formal equivalence between methods to be established. The five-level Tea Root Development Scales (TeaRDS) also provided a practical visual framework for evaluating root development, although further standardization, inter-rater reliability assessment, and external validation are required before its broader use as a phenotyping tool.

Overall, cutting propagation did not provide the same nursery performance as seed propagation in all genotypes; however, selected genotypes achieved comparable developmental profiles under the conditions used in this study. The results provide scientific evidence supporting the potential use of genetically homogeneous and high-quality clonal material, particularly in plantation renewal programs. Future studies evaluating the long-term field performance and economic sustainability of these genotypes under different ecological conditions will further strengthen the feasibility of implementing the proposed production strategies.

## Figures and Tables

**Table 1:** Effects of genotype (G), propagation method (M), and the G × M interaction on growth and quality parameters (G: genotype; M: propagation method; G × M: genotype × propagation method interaction). SD: stem diameter; SQ: shoot quality; RD: root development; SL: shoot length; RL: root length; RW: root weight. Values for SD, SL, RL, and RW are presented as mean ± standard deviation; values for SQ and RD are presented as median [Q1–Q3] (*n* = 3 replicates per genotype × method combination). Effect sizes are given as partial eta-squared (ηp^2^) for the SD, SL, RL, and RW variables, and as epsilon-squared (εr^2^) for the SQ and RD variables. For variables with a significant G × M interaction in ANOVA, means followed by different superscript letters within the same column differ significantly according to Tukey-adjusted multiple comparisons (*p* < 0.05).

Genotype	Method	SD (mm)	SQ (1–5)	RD (1–5)	SL (cm)	RL (cm)	RW (g)
*TUGLALI*	Cutting	2.42 ± 0.13 ^b^	4.83 [4.75–4.92]	4.67 [4.58–4.83]	9.83 ± 0.60	12.10 ± 4.98	0.79 ± 0.04 ^d^
*TUGLALI*	Seed	2.95 ± 0.40 ^ab^	4.50 [4.42–4.75]	4.40 [4.12–4.45]	11.77 ± 0.80	13.73 ± 0.55	1.46 ± 0.13 ^abcd^
*CIN 1*	Cutting	2.85 ± 0.58 ^ab^	4.20 [4.02–4.35]	3.67 [3.58–3.67]	12.07 ± 2.23	12.67 ± 1.75	1.28 ± 0.16 ^bcd^
*CIN 1*	Seed	2.98 ± 0.14 ^ab^	4.83 [4.75–4.92]	4.33 [4.33–4.58]	13.40 ± 1.74	16.37 ± 1.05	1.95 ± 0.28 ^abc^
*SEAKARI*	Cutting	2.82 ± 0.37 ^ab^	3.80 [3.60–4.00]	3.33 [3.27–3.56]	10.40 ± 1.25	13.70 ± 1.31	0.72 ± 0.10 ^d^
*SEAKARI*	Seed	3.07 ± 0.23 ^ab^	4.80 [4.80–4.81]	4.40 [4.29–4.45]	12.80 ± 1.71	14.10 ± 1.37	1.60 ± 0.44 ^abcd^
*53 GU 40*	Cutting	2.71 ± 0.11 ^ab^	4.50 [4.45–4.66]	4.83 [4.81–4.83]	10.73 ± 0.75	15.03 ± 0.65	0.92 ± 0.16 ^d^
*53 GU 40*	Seed	3.36 ± 0.10 ^a^	4.67 [4.67–4.83]	4.50 [4.42–4.58]	15.67 ± 2.48	12.77 ± 0.21	2.02 ± 0.44 ^ab^
*53 ME 265*	Cutting	3.14 ± 0.28 ^ab^	4.67 [4.58–4.69]	4.86 [4.77–4.93]	14.27 ± 1.31	14.00 ± 0.53	1.55 ± 0.37 ^abcd^
*53 ME 265*	Seed	3.14 ± 0.15 ^ab^	4.60 [4.55–4.65]	4.83 [4.83–4.83]	15.13 ± 1.45	14.80 ± 0.79	2.35 ± 0.24 ^a^
*28 TI 09*	Cutting	2.73 ± 0.21 ^ab^	4.67 [4.63–4.69]	5.00 [4.86–5.00]	15.20 ± 1.22	13.77 ± 0.93	1.39 ± 0.25 ^bcd^
*28 TI 09*	Seed	3.42 ± 0.14 ^a^	4.80 [4.54–4.80]	4.33 [4.08–4.50]	16.13 ± 1.52	12.77 ± 1.36	2.03 ± 0.38 ^ab^
*53 CE 279*	Cutting	2.73 ± 0.49 ^ab^	4.33 [4.16–4.56]	4.50 [4.25–4.55]	12.53 ± 0.70	13.63 ± 0.35	1.07 ± 0.19 ^cd^
*53 CE 279*	Seed	3.21 ± 0.14 ^ab^	4.67 [4.63–4.83]	4.67 [4.54–4.75]	16.37 ± 1.25	13.10 ± 0.96	1.88 ± 0.47 ^abc^
*53 AD 83*	Cutting	2.86 ± 0.25 ^ab^	4.50 [4.42–4.75]	4.50 [4.42–4.75]	14.30 ± 2.72	14.27 ± 0.75	1.94 ± 0.09 ^abc^
*53 AD 83*	Seed	2.49 ± 0.03 ^b^	4.60 [4.40–4.70]	4.20 [4.10–4.40]	15.67 ± 0.99	14.80 ± 0.92	1.45 ± 0.46 ^abcd^
*G effect*		F (7, 32) = 2.250 *p* = 0.056; ηp^2^ = 0.330	H (7) = 2.426 *p* = 0.933; εr^2^ = 0.000	H (7) = 18.968 *p* = 0.008; εr^2^ = 0.299	F (7, 32) = 7.467 *p* < 0.001; ηp^2^ = 0.620	F (7, 32) = 0.927 *p* = 0.499; ηp^2^ = 0.169	F (7, 32) = 5.371 *p* < 0.001; ηp^2^ = 0.540
*M effect*		F (1, 32) = 13.724 *p* < 0.001; ηp^2^ = 0.300	H (1) = 4.647 *p* = 0.031; εr^2^ = 0.079	H (1) = 0.603 *p* = 0.437; εr^2^ = 0.000	F (1, 32) = 24.310 *p* < 0.001; ηp^2^ = 0.432	F (1, 32) = 0.813 *p* = 0.374; ηp^2^ = 0.025	F (1, 32) = 55.173 *p* < 0.001; ηp^2^ = 0.633
*G × M interaction*		F (7, 32) = 2.585 *p* = 0.031; ηp^2^ = 0.361	H (7) = 14.803 *p* = 0.039; εr^2^ = 0.195	H (7) = 13.987 *p* = 0.051; εr^2^ = 0.175	F (7, 32) = 1.352 *p* = 0.259; ηp^2^ = 0.228	F (7, 32) = 1.961 *p* = 0.092; ηp^2^ = 0.300	F (7, 32) = 3.856 *p* = 0.004; ηp^2^ = 0.458
*Residual CV (%)*		9.42	-	-	11.44	11.33	19.47

## Data Availability

The data presented in this study is available upon request from the corresponding author.
